# The Role of Physical Activity and Physiotherapists in the Management of Inflammatory Bowel Disease: A Nationwide Cross-Sectional Survey

**DOI:** 10.3390/jcm15083108

**Published:** 2026-04-19

**Authors:** Zita Kovács, Péter Bacsur, Blanka Bernadett Kasza, Ákos Suhajda, Máté Pápista, Noémi Gálfalvi, Ákos Iliás, Bernadett Farkas, Tamás Resál, Klaudia Farkas, Tamás Molnár, Andrea Domján

**Affiliations:** 1 Department of Physiotherapy, Faculty of Health Sciences and Social Studies, University of Szeged, H-6726 Szeged, Hungary; 2 Department of Medicine, Albert Szent-Györgyi Medical School, University of Szeged, H-6725 Szeged, Hungary; 3 Department of Internal Medicine and Oncology, Semmelweis University, H-1088 Budapest, Hungary; 4 Department of Gastroenterology, Central Hospital of Northern Pest-Military Hospital, H-1068 Budapest, Hungary

**Keywords:** physiotherapy, kinesiophobia, inflammatory bowel diseases, physical activity

## Abstract

**Background/Objectives**: Inflammatory bowel diseases (IBDs) cause gastrointestinal symptoms that affect patients’ quality of life. IBD improves with physical activity; however, fear of movement is a limiting factor. This study aimed to evaluate the impact of kinesiophobia and assess patients’ knowledge on the role of physical activity and physiotherapists in IBD management. **Methods**: Participants completed online questionnaires to evaluate demographic and clinical data, lifestyle, physical activity, joint complaints, and physiotherapy preferences. The Tampa Kinesiophobia Scale (TKS) was employed to assess kinesiophobia, and the Godin scale was used to assess regular physical activity. **Results**: Overall, 356 patients with IBD were analyzed. In total, 51% of the patients reported a decrease in physical activity. Of these, 93% have not consulted a physiotherapist, with 51% expressing a need for it. Meanwhile, 75% of the patients wanted additional information. Higher TKS scores were associated with CD, age, and joint pain. The level of kinesiophobia was high and negatively correlated with the amount of physical activity. **Conclusions**: Physiotherapists play an important role in patient education and influencing lifestyle in IBD. Their expertise is underutilized, and patients should be sufficiently informed regarding their illness. Integrating education and physiotherapy may reduce kinesiophobia and improve patients’ quality of life.

## 1. Introduction

Inflammatory bowel disease (IBD) is a chronic and immune-mediated condition characterized by persistent inflammation of the gastrointestinal tract. The two primary forms of IBD are Crohn’s disease (CD) and ulcerative colitis (UC). Although the etiology of IBD is unclear, it is proposed that IBD develops in genetically susceptible individuals exposed to environmental triggers and accompanied by disturbances in the gut microbiota and impaired regulation of the immune system [1]. Notably, extraintestinal manifestations (EIMs) decrease patients’ quality of life (QoL). Musculoskeletal manifestations, which may be axial or peripheral spondyloarthritis (SpA), develop in approximately 40% of patients with IBD, significantly impacting their daily physical activity (PA) [2].

PA improves several chronic conditions, including IBD, by improving QoL, thereby supporting mental health and reducing symptoms [3]. However, symptomatic IBD negatively affects patients’ exercise capacity and participation in daily activities. Approximately 51% of patients reported that IBD has moderately or significantly reduced their fitness levels and their desire to exercise [4]. The PA level varies among patients and is influenced by bowel urgency, toilet access, and medications. Fatigue, anxiety, depression, abdominal pain, and joint pain also reduce PA [5]. Additionally, kinesiophobia—defined as an excessive, irrational, and debilitating fear of physical movement and activity arising from a perceived vulnerability to pain or reinjury—may contribute to reduced physical activity (PA). While this phenomenon has been described in association with chronic pain in several long-term conditions, it has not yet been specifically investigated in inflammatory bowel disease (IBD), despite IBD being a chronic condition in which similar mechanisms may be relevant [6].

Studies recommend low- to moderate-intensity PA for adults with IBD; however, there is no consensus on the form and intensity of the activity. Although 30 days of voluntary exercise can reduce inflammatory responses and decrease the risk of flare-ups, patients often experience a vicious circle between fatigue and exercise, leading to a more sedentary lifestyle. Currently, there are no guidelines regarding the type and intensity of PA in adults with IBD, and Hungarian studies regarding PA in IBD are also lacking [7].

Physiotherapists play an important role in the multidisciplinary care of diseases, including providing education on active living for patients and ensuring appropriate mobility in the event of disease activity, in hospitals and outpatient settings. However, the utilization of physiotherapists in the management of patients with IBD is underutilized as they are often not involved in the multidisciplinary care team [8,9].

Research suggests that the importance of physical activity (PA) is often under-recognized in the management of inflammatory bowel disease (IBD) [10]. Reduced PA may, in part, be attributable to kinesiophobia. The Tampa Scale for Kinesiophobia (TKS), originally developed and validated in populations with musculoskeletal pain, has been widely used to assess fear of movement in the context of pain-related disability. Although kinesiophobia has been primarily studied in musculoskeletal conditions, musculoskeletal manifestations, including joint and muscle pain, are common in IBD [11]. This provides a rationale for extending the assessment of kinesiophobia to IBD, where it may similarly act as a barrier to physical activity and represent a potential target for patient education and behavioral interventions.

Therefore, this study aimed to evaluate the characteristics of patients with IBD in Hungary. Furthermore, we aimed to evaluate the prevalence of kinesiophobia and the knowledge of patients regarding their disease, as well as the role of physiotherapists in IBD management.

## 2. Materials and Methods

### 2.1. Study Design and Participants

In this cross-sectional, nationwide, questionnaire-based study, participants were recruited from Hungary’s largest patient association (Hungarian Crohn’s & Colitis Association), which represents more than 6000 individuals with IBD. The coordinating tertiary IBD hospital was the Centre for Gastroenterology, Department of Medicine, University of Szeged. Adults with IBD were consecutively enrolled between March 2025 and August 2025. Patients with existing comorbidities and history of any traumatic events were excluded. All patients provided written informed consent for regular healthcare and study participation. The reporting of the study conforms to the STROBE guidelines [12].

### 2.2. Data Collection

Data collection was conducted between March 2025 and September 2025. Data were compiled into a standardized Excel database that was stored at the coordinating center. Baseline demographic (age, sex, date of birth, age at diagnosis), and clinical (disease localization by Montreal classification [13], current medications) data were recorded upon study inclusion. PA was evaluated using the Godin Leisure-Time Exercise Questionnaire [14]. The Godin Leisure-Time Exercise Questionnaire is scored by asking how many times per week a person performs strenuous, moderate, and light exercise for at least 15 min. Each frequency is multiplied by a metabolic equivalent (9 for strenuous, 5 for moderate, 3 for light) and summed to produce the total weekly leisure activity score. Higher scores indicate greater levels of physical activity, with scores ≥ 24 typically considered active. Kinesiophobia was assessed by using the original 17-item version of the TKS; each item is rated on a 4-point Likert scale and the total score range was 17–68, with higher scores reflecting greater fear of movement (kinesiophobia) [11,15,16]. We also evaluated PA knowledge and attitudes toward physiotherapists by using a non-validated questionnaire created by physiotherapists and gastroenterologists. The questionnaire is presented in the Supplementary Materials.

### 2.3. Statistical Considerations

A clinical biostatistician was involved in data processing. Sample size calculation was not performed as this was a population-based cross-sectional survey that aimed to include all eligible participants within the study period. Normality was assessed using visual tools. If a normal distribution existed (unless the need to deviate from parametric tests arose), parametric statistical tests were used. Continuous variables are presented as mean + standard deviation (median + IQR if necessary). Discrete variables are presented as frequencies (%). Welch’s two-sample *t*-test was used to test continuous variables, while the Chi-square and Fisher’s exact tests were used for hypothesis testing of discrete variables. Potential associations with categorical variables were analyzed using univariable and multivariable logistic and linear regression models. Specifically, variables with a *p*-value < 0.15 in the univariable analysis were included in the multivariable analysis. Final multivariable models were obtained using forward selection using likelihood ratios. We also performed complete case analysis to reduce bias. Statistical significance was defined as *p* < 0.05, while 95% confidence intervals (95% CI) were communicated. Statistical analysis was performed by using IBM SPSS software (Windows, Version 29.0, IBM Corp., Armonk, NY, USA).

## 3. Results

### 3.1. Patient Characteristics

In total, 356 patients completed the survey (male/female ratio, 40%; median age, 39 years [IQR 31–50]; UC/CD ratio, 41%), of whom 210 (59.0%) had CD. Additionally, 236/356 (66.3%) patients received biological therapy, 231/356 (64.9%) experienced joint pain related to IBD, and 278/356 (76.1%) exercised regularly (walking: 61%; cycling: 38%; ball games: 6%; swimming: 7%; running: 10%). The average duration of PA was 104 (±53) min per week, compared to 109 (±46) min before diagnosis (*p* = 0.92), indicating no measurable decrease in the overall weekly activity time. However, 182/356 patients (51.1%) reported a subjective decline in the intensity of their PA following the diagnosis of IBD. The reasons for the decrease in PA were fatigue (70.0%) and joint pain (40.0%). The demographic, clinical, and PA data are shown in Table 1 and Table 2.

### 3.2. Knowledge and Needs Related to Physiotherapists and Diseases

Overall, 330/356 (92.7%) of patients had never consulted a physiotherapist, and 333/356 (93.5%) had never consulted a trainer during their disease course. Meanwhile, 182/356 (51.1%) of patients agreed that they would need the assistance of a physiotherapist or trainer to develop an appropriate exercise routine. Additionally, 152/356 (42.7%) were unaware of the role of a physiotherapist in patient care, and 196/356 (55.1%) did not know how a physiotherapist could help them manage their disease. Furthermore, 60/356 (16.8%) of patients lacked accurate knowledge regarding their disease, while 95/356 (26.7%) felt that they were not receiving sufficient information about the nature of their disease and other key details. Moreover, 203/356 (55.0%) did not receive adequate information regarding the benefits of exercise. Most respondents (267/356, 75.0%) also expressed a desire to receive additional detailed information about their disease and treatment options, while 231/356 (65.0%) responded that they would be more motivated to engage in exercise if they received reliable and understandable information about the benefits of PA from a professional.

### 3.3. Factors Associated with PA

In our study, the average Godin score was 39.12 (±25.78). Based on the categories derived from Godin scores (Table 3), 249/356 (70%) of participants had an active lifestyle, 53/356 (15%) had a moderately active lifestyle, and approximately 57/356 (16%) had a sedentary lifestyle. Additionally, the amount of PA, based on the Godin score, showed a weak association with the TKS score (*p* = 0.056, B: −0.102, 95% CI for B: −0.79 to −0.01). However, multivariable regression analyses did not reveal any significant correlation between the Godin score and variables such as age, sex, disease type, biological therapy, joint pain, or assistance from a physiotherapist or trainer, and there was no significant association between PA and age, gender, disease type, joint pain, or physiotherapist assistance. Patients receiving biological therapy had a higher rate of regular PA (*p* = 0.058, OR: 1.63, 95% CI: 0.98–2.70), typically with the involvement of a trainer (*p* = 0.053, OR: 7.38, 95% CI: 0.98–55.71). After diagnosis, 182/356 (51.1%) of patients reported a subjective decrease in PA. However, this decrease could not be substantiated by a reduction in the duration of exercise.

### 3.4. Factors Related to Kinesiophobia

**Figure 1:**
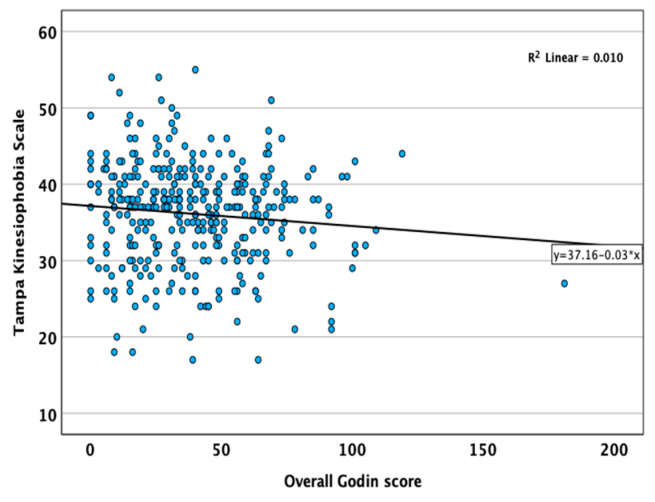
Association between physical activity (Godin scale) and kinesiophobia (TKS).

The average TKS score in our sample was 36.12 (±6.68). Overall, 198/356 (55.6%) of patients had a TKS score > 36. Based on multivariable regression analysis, higher TKS scores were associated with CD (*p* = 0.049, B = −0.101, 95% CI: −2.75 to −0.007), older age (*p* < 0.001, B = 0.26, 95% CI: 0.08 to 0.20), and joint pain (*p* = 0.02, B = 0.12, 95% CI: 0.22 to 3.07). Disease duration showed a non-significant trend toward an association with TKS scores (*p* = 0.07, B = −0.11, 95% CI: −0.16 to 0.01). In contrast, biological therapy, sex, physiotherapist involvement, and trainer participation were not associated with TKS scores. We also confirmed a weak correlation between the Godin and TKS scores (*p* = 0.07, R^2^ = 0.01, Figure 1).

## 4. Discussion

Our analysis revealed a weak inverse correlation between kinesiophobia and the amount of regular exercise. Joint pain was a commonly reported symptom among all participants in our study. More than half of the participants reported a subjective decrease in the intensity of PA after their IBD diagnosis. Although there is a significant need for patient education and physiotherapy care, patients are open to involving a physiotherapist or trainer in the management of their illness. Our results indicate that exercise therapy is still an underutilized resource in everyday IBD management, while PA can have a beneficial effect on physical, psychological, and disease-specific outcomes; these findings are aligned with the literature. Our results also underscore that the lack of information among patients and the underestimation of the role of physiotherapists may be a general problem in IBD management.

In addition to prescribing safe and effective exercise programs, physiotherapists are generally considered to play an important role in monitoring, motivating, and supporting long-term adherence. Their expertise enables the assessment of physical activity, movement patterns, and functional limitations, allowing exercise to be tailored to the patient’s current condition. Furthermore, physiotherapy interventions, including postural correction and breathing exercises, may contribute to improving quality of life and reducing disease burden, as suggested by previous studies [10]. Although modern therapeutic options have significantly improved IBD management over the past decade, PA and a multidisciplinary approach are not given sufficient emphasis in everyday care.

However, these aspects were not directly evaluated in the present study, which focused on patients’ reported use of and perceived need for physiotherapy. Despite advances in medical therapy, physical activity and multidisciplinary approaches may still be underemphasized in routine IBD care.

The incidence of joint pain and inflammation among patients with IBD is between 10% and 30% [3,17], which is lower than that observed in our study (64.9%). This result may be attributed to the high number of patients receiving biological therapy in our study, indicating a more severe disease course, and the self-reported nature of the questionnaire. From a clinical point of view, this result is noteworthy as joint pain and discomfort can cause kinesiophobia, which can result in decreased PA [18]. The prevalence of fatigue is approximately 47% in this population, but it can be as high as 72% during relapses, remarkably exceeding the average value measured in the general population (7%) [19]. Our results are comparable to these values, suggesting that this issue may also play a prominent role in the decrease in PA. Hence, fatigue management should be included in therapy and general patient care. In patients experiencing fatigue and anxiety, patient education is important as engaging in PA can help reduce fatigue. Patients should be encouraged to remain active despite feeling tired as their symptoms are likely to improve with exercise [20].

A significant number of participants had never consulted a physiotherapist, similar to previous studies [21,22,23,24], but there was a demonstrable need for one. In our study, the role of physiotherapists is underestimated from the patients’ viewpoint, presumably because patients are afraid to talk about these issues. Aside from a general lack of knowledge, this may also stem from the need to modify ongoing therapies, as most patients receive treatment in outpatient settings and follow-up in facilities where they do not typically encounter a physiotherapist. Therefore, involving physiotherapists in patient consultations should be considered [22]. Disease-specific fears and beliefs and patients’ lack of information regarding the safe and beneficial framework of PA may also play a role [25]. Regular PA attenuates IBD symptoms, helps achieve and maintain remission, and improves QoL and mental health [26]. Additionally, the attitude of patients with IBD is ambivalent, with many actively avoiding exercise during the active phase of the disease due to fear of worsening symptoms and possible exacerbation [27]. However, our results regarding PA may be overestimated due to response bias as some patients may have refrained from completing the questionnaire due to disease-related fears or a general lack of knowledge.

Kinesiophobia was evaluated using the validated TKS, which has been used in patients with chronic pain [27,28,29]. To the best of our knowledge, our study is the first to apply the TKS in the context of IBD. In other chronic conditions, such as ankylosing spondylitis, the TKS has demonstrated a clear correlation with lower PA levels and greater functional limitations [30,31]. There is a negative correlation between kinesiophobia and PA, and education and the promotion of exercise as well as targeted exercise programs are recommended as solutions to reduce the irrational fear [32]. Notably, our results are comparable to the international average TKS score of 37 [33,34]. PA was assessed using the Godin Leisure-Time Exercise Questionnaire, whose classification rules and validity are widely documented and which is based on self-reporting and requires subjective assessment of intensity [14]. In our study, a weak association between kinesiophobia and Godin scores was observed; however, this did not reach statistical significance and should be interpreted with caution, although it may still have potential clinical relevance [30,35]. The assessment of kinesiophobia may be integrated into gastroenterological outpatient care to support targeted patient education and individualization of physiotherapy programs according to the principle of gradual progression. Due to the cross-sectional design of our study, no causal relationship can be established, as higher kinesiophobia may be a consequence and a maintaining factor of reduced PA. Furthermore, the TKS was originally developed for chronic musculoskeletal pain, with items focusing on fears of injury and physical harm. Its application in IBD may therefore introduce a degree of construct mismatch, as patients with IBD may avoid physical activity due to concerns about triggering gastrointestinal symptoms (e.g., pain, urgency, or incontinence) rather than injury. This discrepancy may limit the construct validity of the TKS in this population, and our findings should be interpreted accordingly.

The strength of our study lies in its nationwide setting and large number of participants. Another strength is the use of standardized and validated measurement tools for key outcomes (TKS and Godin Leisure-Time Exercise Questionnaire), and the choice of a topic that fills a gap in the literature. To our knowledge, we have not encountered any publication that evaluates the degree of kinesiophobia in IBD. Our limitations include the use of a non-validated questionnaire to assess exploratory/descriptive data (knowledge related to physiotherapists and the disease), and although the Godin questionnaire is validated, it is self-reported, which may also distort the data. As this was a questionnaire-based study, subtle nuances in language and phrasing may have introduced subjectivity, potentially influencing the consistency of the collected data. Furthermore, patient recruitment and collecting responses via an online platform may have introduced selection bias. A precise response rate could not be determined due to recruitment via online community groups, where the total size of the target population was unknown. This methodological characteristic limits the reliable estimation of the response rate and, consequently, the ability to assess the extent of potential selection bias. Another limitation is the relatively high proportion of patients receiving biologic therapy, reflecting a more severe disease course. Moreover, as patient-reported data were used, the classification of joint pain as an extraintestinal manifestation was not clearly defined, whereas response and selection bias are high. Disease activity data were not collected in this study due to the lack of consistently documented and standardized measures across the study population, as well as the limited availability of complete clinical records for all participants. This represents a limitation, as the absence of disease activity information precludes the assessment of its potential influence on the observed outcomes. The retrospective question regarding “prior regular physical activity” is subject to recall bias, which may affect the accuracy of the responses. In addition, IBD is a fluctuating condition, meaning that kinesiophobia and physical activity levels may vary between flare and remission periods, which cannot be fully captured by a single-time-point assessment. We did not include a comparison with the general Hungarian population regarding physical activity levels, as no standardized, nationally representative data are currently available. Furthermore, socioeconomic and educational variables were not assessed, although these factors may influence health behaviors, including PA. These factors should be considered when interpreting the results, and longitudinal studies are recommended to more accurately track changes over time.

In conclusion, we suggest that monitoring joint pain, kinesiophobia, regular PA, and general fatigue may be considered as part of outpatient care consultations, including within physiotherapy settings. Patient education delivered by physiotherapists may help ensure that the type and intensity of exercise performed are adapted to the intermittent symptoms of the disease. Engaging physiotherapists, creating tailored exercise programs, and implementing structured educational strategies may represent potential approaches to improving patients’ PA and overall well-being. Multidisciplinary care and cooperation between specialists (gastroenterologist, physiotherapist, psychologist, rheumatologist, and dietitian) may be beneficial in the management of patients with joint complaints, fatigue, or kinesiophobia. In the future, prospective multicenter studies that employ objective activity meters to measure regular PA are warranted.

## Figures and Tables

**Table 1:** Demographic and clinical characteristics of enrolled participants.

Variables	IBD (*n* = 356)
**Disease type,** *CD, n (%)*	210 (59.0)
**Sex,** *male (%)*	142 (39.9)
**Age at inclusion**, *years, median (IQR)*	39.0 (31–50)
**Disease duration at inclusion,** *years, median (IQR)*	12.0 (6–20)
**Biological treatment use,** *n (%)*	236 (66.3)
**Arthritis, arthralgia present,** *n (%)*	231 (64.9)

Abbreviations: IBD: Inflammatory bowel disease; CD: Crohn’s disease; *n*: number of patients; IQR: inter-quartile range.

**Table 2:** Exercise behavior and level of kinesiophobia of enrolled participants.

Variables	IBD (*n* = 356)
**Prior regular physical activity,** *n (%)*	271 (76.1)
**Recent regular physical activity,** *n (%)*	270 (75.8)
**Guidance,** *n (%)*	49 (13.8)
Physiotherapist	26 (7.3)
Trainer	23 (6.5)
**Godin score,** *mean (±SD)*	39.1 (25.8)
**TKS score,** *mean (±SD)*	36.1 (6.7)

Abbreviations: IBD: Inflammatory bowel disease; *n*: number of patients, SD: standard deviation, TKS: Tampa Kinesiophobia Scale.

**Table 3:** Details of Godin scale for measuring physical activity.

Godin Scale Score	Interpretation	*n* (%)
24 points or more	Active lifestyle	248 (69.7)
14–23 points	Moderately active lifestyle	52 (14.6)
Less than 14 points	Insufficient activity/sedentary lifestyle	56 (15.7)

Abbreviations: *n*: number of patients.

## Data Availability

A.D. is the guarantor of the article. Original data regarding the manuscript are not available. The current manuscript, including related data and figures, has not been previously published and is not under consideration elsewhere.

## Supplementary Materials

The following supporting information can be downloaded at: https://www.mdpi.com/article/10.3390/jcm15083108/s1.

## Abbreviations

The following abbreviations are used in this manuscript:

TKS	Tampa Kinesiophobia Scale
IBD	inflammatory bowel disease
CD	Crohn’s disease
*n*	number of patients
IQR	interquartile range
PA	physical activity
CI	confidence intervals
EIMs	extraintestinal manifestations
