# Supplementary material for: The Role of Physical Activity and Physiotherapists in the Management of Inflammatory Bowel Disease: A Nationwide Cross-Sectional Survey
Source: J Clin Med. 2026 Apr 19;15(8):3108. doi: 10.3390/jcm15083108 (PMC13117172; doi:10.3390/jcm15083108)
Supplement: Supplementary file 1 [file jcm-15-03108-s001.zip › jcm-4204878-supplementary.pdf]

Questionnaire

Disease characteristics

Type of disease:
- Crohn's disease
- Ulcerative colitis

Age:

Sex:
- Male
- Female

Year of diagnosis:

Are you currently receiving biological therapy?
- Yes (Zessly, Inflectra, Hukyndra, Hyrimoz, Yuflyma, Amgevita, Stelara, Entyvio, Xeljanz, Rinvoq, Zeposia)
- No

Have you experienced joint pain related to your disease?
- Yes
- No

Do you currently engage in regular physical activity?
- Yes
- No

If yes, what type of physical activity do you perform regularly?
- Walking
- Cycling
- Swimming
- Running
- Ball sports
- Other:

Approximately how many minutes per week do you spend on physical activity?
- <15 minutes
- 15–30 minutes
- 30–60 minutes
- 60–90 minutes
- 90–150 minutes
- >150 minutes

Did you perform regular physical activity before being diagnosed with IBD?
- Yes
- No

If yes, how many minutes per week did you spend on physical activity?
- <15 minutes
- 15–30 minutes
- 30–60 minutes
- 60–90 minutes
- 90–150 minutes
- >150 minutes

Have you been less physically active since your diagnosis?
- Yes
- No

If yes, what do you think is the reason?
- Fatigue
- Worsening of symptoms
- Did not see the benefit
- Did not feel any positive effect
- Other:

In your opinion, how does regular physical activity affect IBD symptoms?
- Clearly worsens
- Rather worsens
- No effect
- Rather improves
- Clearly improves

How does physical activity help you cope with your disease?
- Improves well-being
- Reduces fatigue
- Helps with daily activities
- Reduces symptoms
- Other:

Have you received support from a trainer related to your disease?
- Yes
- No

Have you received support from a physiotherapist?
- Yes
- No

I would like support from a physiotherapist/trainer to develop appropriate physical activity habits:
- Strongly disagree
- Disagree
- Undecided
- Agree
- Strongly agree

I am familiar with the role of a physiotherapist in patient care:
- Strongly disagree
- Disagree
- Undecided
- Agree
- Strongly agree

I understand how a physiotherapist can help in coping with my disease:
- Strongly disagree
- Disagree
- Undecided
- Agree
- Strongly agree

I have sufficient knowledge about my disease:
- Strongly disagree
- Disagree
- Undecided
- Agree
- Strongly agree

I feel that I receive adequate information about my disease:
- Strongly disagree
- Disagree
- Undecided
- Agree
- Strongly agree

I feel that I receive adequate information about the benefits of physical activity:
- Strongly disagree
- Disagree
- Undecided
- Agree
- Strongly agree

I would like more detailed information about my disease and its treatment:
- Strongly disagree

- Disagree
- Undecided
- Agree
- Strongly agree

If I received adequate information from a professional about the benefits of physical activity, I would be more motivated to exercise:
- Strongly disagree
- Disagree
- Undecided
- Agree
- Strongly agree

During a typical week, how many times do you perform the following activities for more than 15 minutes?

Vigorous physical activity (rapid heart rate): ____ times/week
(e.g., running, football, basketball, skating, intensive swimming)

Moderate physical activity: ____ times/week
(e.g., brisk walking, cycling, tennis, light swimming)

Light physical activity: ____ times/week
(e.g., yoga, walking, golf)

During a typical week, how often do you perform physical activity that causes sweating or increased heart rate?
- Often
- Sometimes
- Rarely/Never

Tampa Scale for Kinesiophobia (TSK)

1 = strongly disagree
2 = disagree
3 = agree
4 = strongly agree

I am afraid that I might injure myself if I exercise
1 2 3 4

If I tried to overcome it, my pain would increase
1 2 3 4

My body is telling me something is seriously wrong
1 2 3 4

If I exercised, my pain would probably decrease
1 2 3 4

People do not take my medical condition seriously enough
1 2 3 4

My body is at risk for the rest of my life because of my condition
1 2 3 4

Pain always means that my body is injured
1 2 3 4

If something increases my pain, it does not necessarily mean it is dangerous
1 2 3 4

I am afraid that I might accidentally injure myself
1 2 3 4

The safest way to prevent pain is to avoid unnecessary movements
1 2 3 4

My pain would not be so severe if nothing dangerous were happening in my body
1 2 3 4

Although my condition is painful, I would be better off if I were physically active
1 2 3 4

Pain signals me to stop exercising to avoid injury
1 2 3 4

It is not really safe for a person in my condition to be physically active
1 2 3 4

I cannot do everything that normal people do because I am too easily injured
1 2 3 4

Even if something causes significant pain, it does not necessarily mean it is dangerous
1 2 3 4

No one with pain should exercise
1 2 3 4

Thank you for completing the questionnaire!

Kérdőív

1. **Betegség típusa:**

☐ Crohn betegség ☐ Colitis ulcerosa

2. **Életkor:**
3. **Nem:**

☐ Férfi ☐ Nő

4. **Diagnózis éve:**
5. **Jelenleg biológiai terápiában részesülök:**

☐ Igen (Zessly, Inflectra, Hukyndra, Hyrimoz, Yuflyma, Amgevita, Stelara, Entyvio, Xeljanz, Rinvoq, Zeposia) ☐ Nem

6. **Betegségével összefüggésben tapasztalt-e ízületi fájdalmat?**

☐ Igen ☐ Nem

7. **Jelenleg végez-e rendszeresen testmozgást?**

☐ Igen ☐ Nem

8. **Ha igen, milyen rendszeres testmozgást végez?**

☐ gyaloglás ☐ biciklizés ☐ úszás ☐ futás ☐ labdajátékok **..............** egyéb

9. **A heti rendszeres testmozgás körülbelül hány percet vesz igénybe egy hét alatt összesen?**

☐ 15 percnél kevesebb ☐ 15-30 perc ☐ 30-60 perc ☐ 60-90 perc ☐ 90-150 perc ☐ 150 percnél több

10. **A gyulladásos bélbetegség diagnózisa előtt végzett-e valamilyen testmozgást?**

☐ Igen ☐ Nem

11. **Ha végzett, akkor a heti rendszeres testmozgás körülbelül hány percet vett igénybe egy hét alatt összesen?**

☐ 15 percnél kevesebb ☐ 15-30 perc ☐ 30-60 perc ☐ 60-90 perc ☐ 90-150 perc ☐ 150 percnél több

12. **A gyulladásos bélbetegség diagnózisa óta kevesebb testmozgást végez?**

☐ Igen ☐ Nem

13. Ha igen, mit gondol, mi lehet ennek a hátterében?

□ fáradtság miatt
□ a tüneteim romlottak
□ nem láttam értelmét
□ nem éreztem jótékony hatását
egyéb ....

14. Ön szerint milyen hatással van a rendszeres időközönként végzett aktív testmozgás az IBD tüneteire?

□ egyértelműen rontja
□ inkább rontja
□ nincs hatással
□ inkább javítja
□ egyértelműen javítja

15. Ön szerint min/hogyan segít a testmozgás a betegséggel való megküzdés során?

□ javítja a közérzetem
□ kevésbé leszek fáradékony
□ könnyebben ellátom a mindennapi teendőket
□ csökkenti a tüneteimet
egyéb ....

16. A bélbetegséggel kapcsolatban edzői segítséget igénybe vett/vesz-e?

□ Igen
□ Nem

17. A betegséggel kapcsolatban gyógytornász segítséget igénybe vett/vesz-e?

□ Igen
□ Nem

18. Lenne rá igényem, hogy gyógytornász/edző segítsen a megfelelő testmozgás kultúra kialakításában:

□ egyáltalán nem értek egyet
□ inkább nem értek egyet
□ nem tudom
□ inkább egyetértek
□ teljesen egyetértek

19. Ismerem, hogy egy gyógytornász milyen feladatokat lát el a betegellátásban:

□ egyáltalán nem értek egyet
□ inkább nem értek egyet
□ nem tudom
□ inkább egyetértek
□ teljesen egyetértek

20. Ismerem, hogyan segíthet egy gyógytornász a betegséggel való megküzdésben:

□ egyáltalán nem értek egyet
□ inkább nem értek egyet
□ nem tudom
□ inkább egyetértek
□ teljesen egyetértek

21. Pontos ismereteim vannak a betegségemről:

□ egyáltalán nem értek egyet
□ inkább nem értek egyet
□ nem tudom
□ inkább egyetértek
□ teljesen egyetértek

22. Úgy érzem megkapom a kellő információt a betegségemmel kapcsolatban:

☐	☐	☐	☐	☐
egyáltalán nem értek egyet	inkább nem értek egyet	nem tudom	inkább egyetértek	teljesen egyetértek

23. Úgy érzem megkapom a kellő információt a testmozgás hatékonyságával kapcsolatban:

☐	☐	☐	☐	☐
egyáltalán nem értek egyet	inkább nem értek egyet	nem tudom	inkább egyetértek	teljesen egyetértek

24. Lenne igényem arra, hogy részletesebb tájékoztatást kapjak a betegségemmel és annak kezelésével kapcsolatban

☐	☐	☐	☐	☐
egyáltalán nem értek egyet	inkább nem értek egyet	nem tudom	inkább egyetértek	teljesen egyetértek

25. Ha egy szakembertől megkapnám a kellő tájékoztatást a testmozgás jótékony hatásairól, több kedvem lenne mozogni:

☐	☐	☐	☐	☐
egyáltalán nem értek egyet	inkább nem értek egyet	nem tudom	inkább egyetértek	teljesen egyetértek

Egy átlagos **7 napos időtartam** (egy hét) alatt átlagosan hány alkalommal végzi szabadidejében **15 percnél hosszabb ideig** a következő testmozgástípust (írja minden vonalra a megfelelő számot).

a) MEGERŐLTETŐ TESTMOZGÁS (GYORS SZÍVVERÉS)
heti__________ alkalommal
(pl.: futás, kocogás, hoki, labdarúgás, amerikai futball, squash, kosárlabda, sífutás, judo, görkorcsolya, erőteljes úszás, erőteljes hosszú távú kerékpározás)

b) KÖZEPES ERŐSSÉGŰ TESTMOZGÁS (NEM MEGTERHELŐ)
heti _________ alkalommal
(pl.: tempós gyaloglás, baseball, tenisz, könnyed kerékpározás, kézilabda, röplabda, tollas, könnyed úszás, alpesi síelés, tánc)

c) KÖNNYŰ TESTMOZGÁS (MINIMÁLIS MEGERŐLTETÉS)
heti __________ alkalommal
(pl.: jóga, íjászat, folyami horgászat, bowling, golf, hómobil, könnyed séta)

Egy átlagos **7 napos időtartam** (egy hét) alatt átlagosan milyen gyakran végez olyan mértékű rendszeres testmozgást szabadidejében, **amely megizzasztja** (gyors szívverés)?

GYAKRAN ☐ NÉHA ☐ SOHA/RITKÁN ☐

Tampa Kineziofóbia Skála (karikázza be az Ön véleményét leginkább jellemző pontszámot)

1= határozottan nem ért egyet **2**= nem ért egyet **3**= egyetért **4**= határozottan egyetért

1.	Félek, hogy megsérülhetek, ha tornázom	1	2	3	4
2.	Ha megpróbálnék túllendülni rajta, akkor a fájdalmam erősödne	1	2	3	4
3.	A testem azt üzeni, hogy valami veszélyes bajom van	1	2	3	4
4.	Ha tornáznék, valószínűleg csökkenne a fájdalmam	1	2	3	4
5.	Az emberek nem veszik elég komolyan az egészségi állapotomat	1	2	3	4
6.	Az életem hátralévő részére a testem veszélybe került a sérülésem miatt	1	2	3	4
7.	Számomra a fájdalom mindig azt jelenti, hogy megsérült a testem	1	2	3	4
8.	Ha valami súlyosbítja a fájdalmam, az nem feltétlen jelenti azt, hogy az veszélyes is	1	2	3	4
9.	Félek attól, hogy véletlenül sérülést okozok magamnak	1	2	3	4
10.	A fájdalmam súlyosbodásának elkerülésére a legbiztosabb dolog, amit tehetek, ha egyszerűen óvatos vagyok, hogy ne tegyek felesleges mozdulatokat	1	2	3	4
11.	Nem lenne ekkora a fájdalmam, ha nem zajlana valami potenciálisan veszélyes folyamat a testemben	1	2	3	4
12.	Bár az állapotom fájdalommal jár, mégis jobban lennék, ha fizikailag aktív lennék	1	2	3	4
13.	A fájdalom jelzi számomra, hogy hagyjam abba a tornázást, hogy ne sérüljek meg	1	2	3	4
14.	Egy olyan állapotban lévő személynek, mint én, nem igazán biztonságos, hogy fizikailag aktív legyen	1	2	3	4
15.	Nem tudok mindent megtenni, amit a normális emberek tesznek, mert túl könnyen megsérülök	1	2	3	4
16.	Annak ellenére, hogy valami nagy fájdalmat okoz nekem, nem gondolom, hogy ez valójában veszélyes	1	2	3	4
17.	Senkinek nem kellene tornáznia, akinek fájdalma van	1	2	3	4

Köszönjük a kitöltését!
